# Exosomes in Coronary Artery Disease

**DOI:** 10.7150/ijbs.36427

**Published:** 2019-09-07

**Authors:** Xiao-Fei Gao, Zhi-Mei Wang, Feng Wang, Yue Gu, Jun-Jie Zhang, Shao-Liang Chen

**Affiliations:** 1 Department of Cardiology, Nanjing First Hospital, Nanjing Medical University, Nanjing, China;; 2 Department of Cardiology, Nanjing Heart Centre, Nanjing, China.; The first two authors contributed equally to this work.

**Keywords:** extracellular vesicles, exosomes, coronary artery disease

## Abstract

Exosomes, the nanosized vesicles released from various cell types, contain many bioactive molecules, such as proteins, lipids, and nucleic acids, which can participate in intercellular communication in a paracrine manner or an endocrine manner, in order to maintain the homeostasis and respond to stress adaptively. Currently, exosomes have already been utilized as diagnostic biomarkers and therapeutic tools in cancer clinical trials. There has also been great progress in cell and animal exosomes studies of coronary artery disease (CAD). Emerging evidence suggests that exosomes released from endothelial cells, smooth muscle cells, adipose cells, platelets, cardiomyocytes, and stem cells have been reported to play crucial roles in the development and progression of CAD. Moreover, it has been showed that exosomes released from different cell types exhibit diverse biological functions, either detrimental or protective, depending on the cell state and the microenvironment. However, the systematic knowledge of exosomes in CAD at the patient level has not been well established, which are far away from clinical application. This review summarizes the basic information about exosomes and provides an update of the recent findings on exosome-mediated intercellular communication in the development and progression of CAD, which could be helpful for understanding the pathophysiology of CAD and promoting the further potential clinical translation.

## Introduction

Coronary artery disease (CAD) is a common condition that strongly correlates with increased cardiovascular morbidity and mortality, and it poses a high economic burden to the national healthcare system 1,2. An overall better understanding of the mechanisms of atherosclerosis, the development of antiplatelet and statin therapies, and advances in devices and procedural techniques for coronary intervention have contributed to a great improvement in the clinical prognosis of patients 3. Despite this, patients with coronary disease still face the residual risks of stent restenosis, cardiac remodeling, and ischemia/reperfusion injury. Thus, further exploration of the pathophysiology of CAD is important, and a novel, reliable tool for these patients will be of great interest.

Extracellular vesicles, membrane-bound organelles and released by diverse cell types, contain many bioactive molecules, such as proteins, lipids, and nucleic acids; these vesicles are varied in size, biogenesis and contents, which usually include apoptotic bodies, microvesicles (also called ectosomes, microparticles, or matrix vesicles), and exosomes 4,5. The similarities and differences of these three vesicles are summarized in **Table 1**. In brief, apoptotic bodies are large vesicles (more than 1 µm in diameter) with a permeable membrane that are formed upon cell apoptosis 5,6. Microvesicles (0.1-1 µm in diameter) are generated by shedding directly from the plasma membrane and undergo the process of cytoskeleton remodeling and the externalization of phosphatidylserine 5,7. Exosomes are the smallest vesicles (30-150 nm in diameter) of endosomal origin and are released into extracellular space upon the fusion of multivesicular bodies (MVBs) with the plasma membrane, which occurs in an endosomal sorting complex required for transport (ESCRT)-dependent or ESCRT-independent manner 7-9.

In the past decade, emerging evidence in the field of cardiovascular disease 10-16 reported that exosomes play crucial roles in intercellular communication via transferring cargo (including proteins, RNA, DNA, and other molecules) under not only physiological situations (angiogenesis and cardiac growth) but also pathological conditions (ischemia/reperfusion injury, vascular calcification, atherosclerosis, and cardiac remodeling). In this review, we focus on the updated findings in exosomes biogenesis, secretion and uptake, isolation and detection, as well as exosomes-mediated intercellular communication in the development and progression of CAD.

## Exosomes Biogenesis, Secretion and Uptake

**Figure 1:**
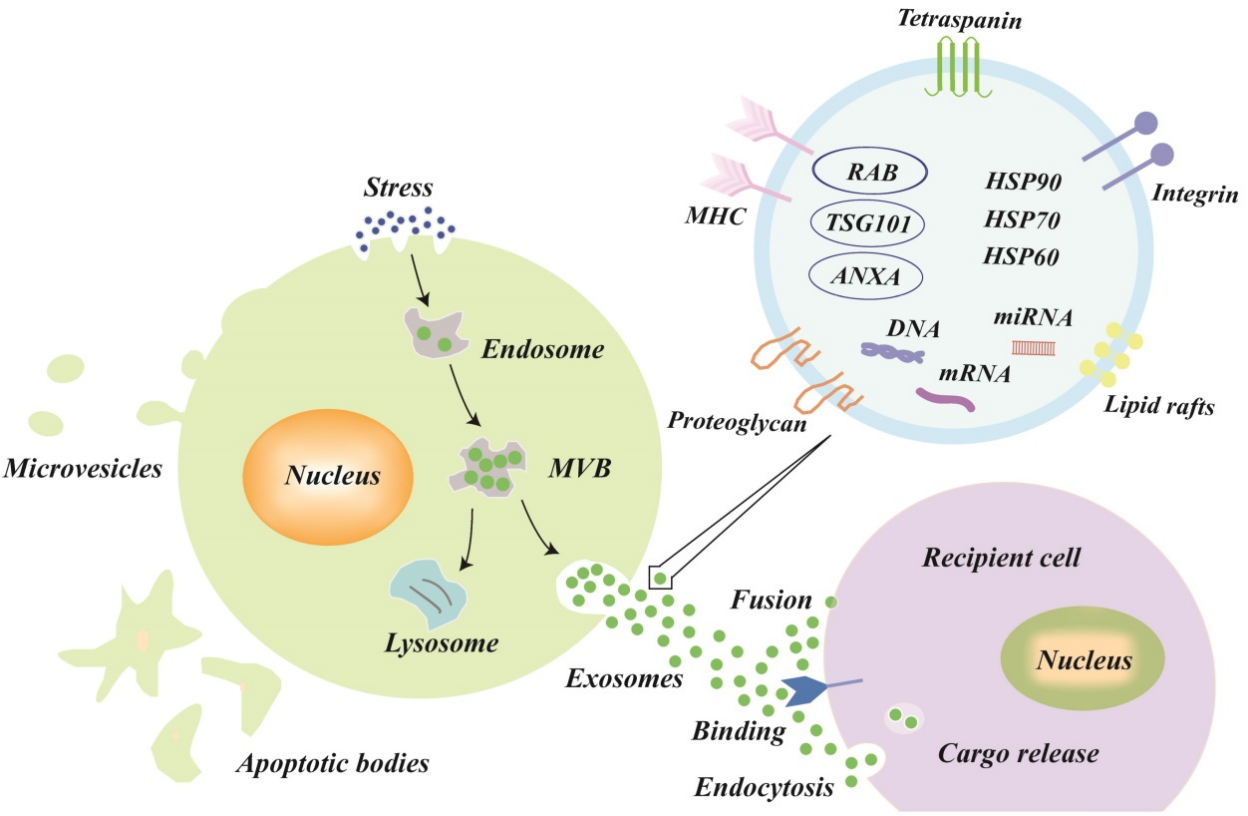
** Biogenesis, secretion, uptake and the schematic presentation of exosomes.** Endosomes are formed by the inward budding of the cell membrane. Multivesicular bodies (MVBs) originate in the lumen of the endosomes by an inward budding of the endosomal membrane, which has two outcomes: either fusing with the plasma membrane to release its intraluminal vesicles to the extracellular space (called exosomes) or fusing with lysosomes for lysosomal degradation. The three types of mechanisms of exosome uptake by recipient cells are fusion, binding, and endocytosis. In general, the exosomes are formed with the following molecules: tetraspanin family (CD9, CD63, and CD81), lipid rafts, integrin, tumor susceptibility gene 101 (TSG101), major histocompatibility complex (MHC) class II molecules, heat shock proteins (HSP60, HSP70, and HSP90), annexins, and nucleic acids.

The explicit mechanisms of exosomes biogenesis are quite complicated and only partly understood 17,18. In brief, early endosomes, which are formed by the inward budding of the cell membrane, can mature into late endosomes 19,20. Intraluminal vesicles (ILVs), also called exosomes precursors, originate in the lumen of late endosomes by the inward budding of the endosomal membrane 21. The late endosomes that contain ILVs become MVBs, which have two possible outcomes 22: either fusing with the plasma membrane to release the ILVs to the extracellular space (called exosomes) or fusing with lysosomes for lysosomal degradation (**Figure 1**).

Several key molecules have been verified to participate in membrane fusion and the release of exosomes into the extracellular space. Small Rab GTPases were identified to promote exosomes secretion, and Rab27a and Rab27b were involved in MVB docking at the plasma membrane 23. Hyenne et al 24 also found that RAL-1 (Ras-related GTPase homolog) controlled MVB formation and exosomes secretion. Moreover, soluble N-ethylmaleimide-sensitive factor attachment protein receptors (SNAREs) are important molecules for vesicle fusion 6,25-27. Recently, Zou et al 28 found that exosomes release, such as autophagy, could be regulated by the mechanistic target of rapamycin complex 1 (mTORC1) in response to changes in amino acids and growth factor conditions, both in cultured cells and *in vivo*.

After secretion, exosomes in the extracellular space can be taken up by recipient cells to affect the gene expression and function of recipient cells. There are three known types of exosomes uptake mechanisms as follows: fusion, binding, and endocytosis (**Figure 1**) 29,30. After exosomal membranes directly fuse with recipient cell membranes, the cargo, including proteins, miRNAs, mRNAs, etc. are released into the cytoplasm of recipient cells. Exosomes, with external ligands 31, can also bind to the receptors of recipient cells to activate signal transduction. Moreover, the mechanisms of endocytosis 9,30,32,33 (the most frequent type) vary greatly and include macropinocytosis, phagocytosis, caveolae-mediated endocytosis, lipid raft-mediated endocytosis, and clathrin-mediated endocytosis; the type of endocytosis may depend on the cell types and physiological conditions. For example, exosomes uptake may occur via extracellular signal-regulated kinase 1/2 (ERK 1/2) and heat shock protein 27 (HSP27) signaling or lipid raft-mediated endocytosis, which was negatively regulated by caveolin-1 during exosomes internalization 34.

## Exosomes Isolation and Detection

The current known contents of exosomes include at least 41,860 proteins, more than 7,540 RNAs and 1,116 lipid molecules 35, and the content of the exosomes varies greatly according to their parent cell type and microenvironment 36-40. In general, the exosomes are formed with the following molecules: tetraspanin family (CD9, CD63, and CD81 (transmembrane proteins)), tumor susceptibility gene 101 (TSG101, used for sorting and transporting exosomes), major histocompatibility complex (MHC) class II molecules, and programmed cell death 6-interacting proteins (PDCD6IPs) 18,22,37,38,41. Additionally, heat shock proteins (HSP60, HSP70, and HSP90), cytoskeletal proteins (actin and tubulin), annexins (regulate cytoskeletal changes in membranes and membrane fusion), and membrane transport proteins are also present in exosomes (**Figure 1**), irrespective of the type 18,22,38,41.

Exosomes could be isolated from body fluids or cell culture media by several different techniques, including differential and gradient density centrifugation, filtration, immunological separation, precipitation, and commercial exosomes isolation kits 18,41,42. Briefly, centrifugation-based techniques are the most common method to isolate exosomes, and this method is also regarded as the gold standard for exosomes isolation 43, but this method has the following limitations: a large sample volume, time-consuming, and a low exosomes recovery (5%-25%) 44-46. Filtration-based techniques are currently used to eliminate dead cells and debris, which prepares the sample for further ultracentrifugation 18,47. Immunological separation has a high specificity because antibody-coated magnetic beads bind to the proteins in the exosomes, but this technique was blamed for a high cost and a low exosome yield 46,48. The exosome precipitation method is utilized in several commercial exosomes isolation kits and has many advantages, including simplicity, low sample volume, and cost-effectiveness 47,49. However, the precipitated samples are mixed with contaminants (proteins, lipids, etc.), which are not suitable for direct further detection accurately. Of note, a recent study 50 found that different cell culture conditions and isolation methods could result in varying glycosylated exosomes populations. Overall, the selection of isolation method should be based on the down-stream application and the experiment purpose.

The morphology of exosomes could be detected by a variety of electron microscopy techniques, including transmission electron microscopy (TEM), scanning electron microscopy, cryo-electron microscopy and atomic force microscopy 18. Western blots and (high resolution) flow cytometry are widely used to identify the specific surface protein markers of exosomes. The size of the exosome is measured by nanoparticle tracking analysis (NTA), tunable resistive pulse sensing, and dynamic light scattering 22,51. In the current basic research on exosomes, combinations of TEM, western blots (or flow cytometry), and NTA are the most widely used tools to detect exosomes. Moreover, according to minimal experimental requirements for the definition of extracellular vesicles from the International Society for Extracellular Vesicles 22,41, exosomes are defined as vesicles that contain at least one transmembrane protein (CD9, CD63, CD81, adhesion molecules, etc.) and one cytosolic protein (TSG101, annexins, Rabs, etc.), without any endoplasmic reticulum proteins (calnexin and Golgi matrix proteins) or nuclear proteins.

Several key points are quite crucial for the exosome isolation and detection. To exclude the effects of exosomes in fetal bovine serum (FBS), more than 100,000 x g ultracentrifugation of complete medium (or of FBS following at least 1:4 dilution) for at least 18 hours should be performed 41. Commercial exosomes-depleted FBS is also available from vendors. Additionally, the storage temperature of exosomes is very important. Exosomes storage at -80℃ for three to six months could maintain the basic functions, but current view suggests that original samples are stored at -80℃ and exosomes are isolated just before the index experiments 41,52-54.

## Exosomes and Coronary Artery Disease

**Figure 2:**
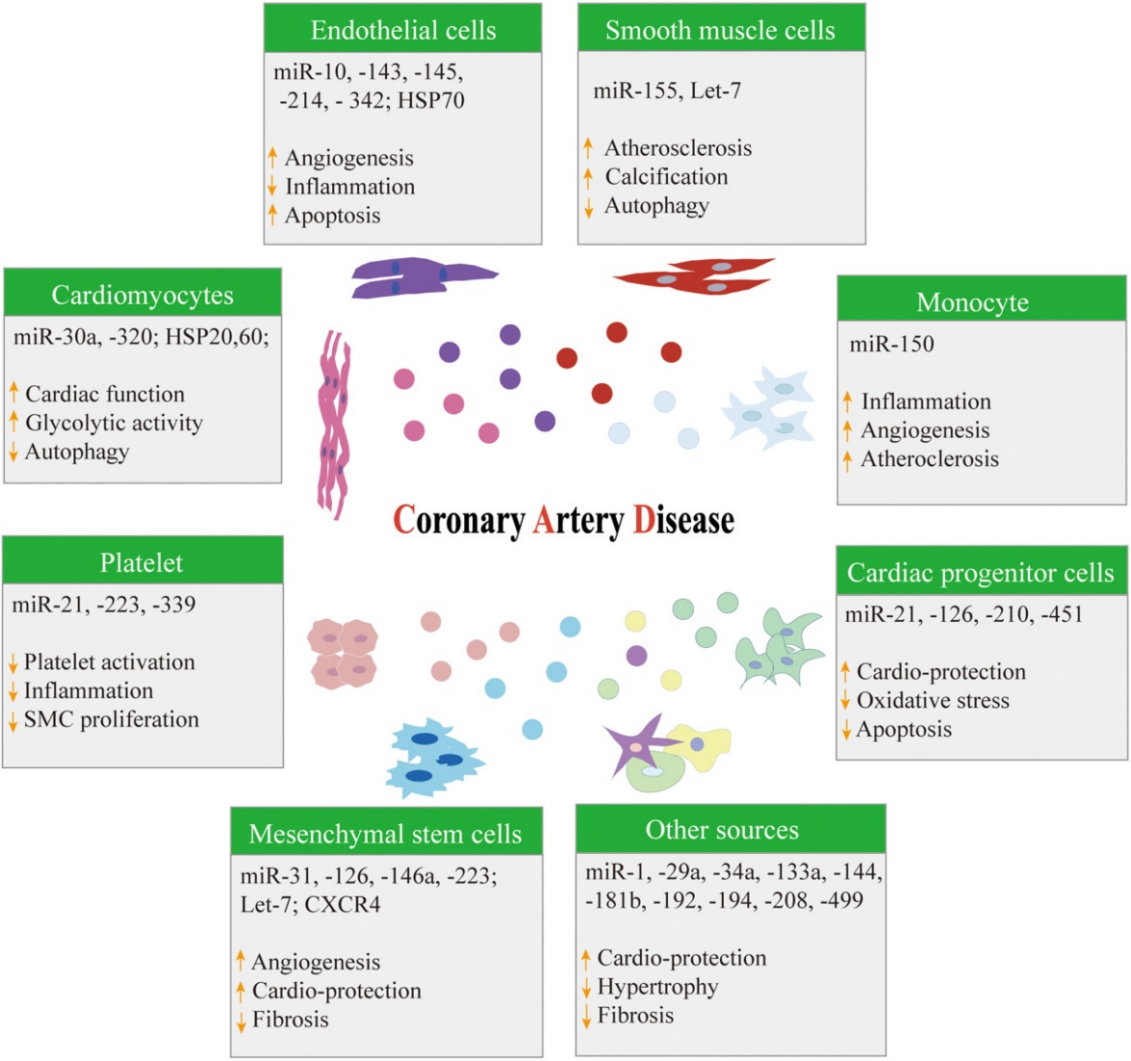
** A summary of different cell-derived exosomes in coronary artery disease.** HSP: Heat shock protein; SMC: Smooth muscle cell; CXCR4: C-X-C chemokine receptor type 4.

The specific mechanism of atherosclerosis is complex, and the mechanism is a chronic process with multifactorial causes. The current view 55-58 is that oxidative stress, endothelial dysfunction, and inflammation are three key factors involved in the development and progression of atherosclerosis. Exosomes participate in cardiovascular cell-to-cell (paracrine) and distant (endocrine) communication via miRNAs and other mediators 59. Exosomes released from stem cells, endothelial cells, smooth muscle cells, cardiomyocytes, adipose cells, and platelets include potential valuable biological information for the development and progression of CAD (**Figure 2**) 5.

### Cardiac progenitor cell-derived exosomes

Exosomes released by cardiac progenitors have been shown to prevent ischemic myocardium against acute ischemia/reperfusion injury through miR-451 60. Cardiac progenitor cell-derived exosomes could also protect cardiomyocytes against oxidative stress-related apoptosis via exosomal miR-21 by targeting programmed cell death 4 (PDCD4) 61. Moreover, the exosomal transfer of miR-126 and miR-210 from host cells to transplanted cells could improve the survival of transplanted cardiac progenitor cells into the ischemic myocardium 62.

### Mesenchymal stem cell-derived exosomes

Exosomes derived from mesenchymal stem cells (MSCs) improved atherosclerosis in ApoE^-/-^ mice and promoted M2 macrophage polarization in the atherosclerotic plaque by the microRNA-let7/HMGA2/NF-κB pathway 63. Kang et al 64 also reported that C-X-C chemokine receptor type 4 (CXCR4)-overexpressing MSC-derived exosomes could reduce infarct size, improve cardiac remodeling, and increase angiogenesis by activating the Akt signaling pathway following myocardial infarction. Furthermore, exosomes released from MSCs regulated the protein expression of adherent junctions, leading to angiogenesis via miRNA 31 65, miRNA 126 65, miRNA 146a 66, and miRNA 223 67.

### Endothelial cell-derived exosomes

Endothelial cell-derived exosomes can reduce ischemia and reperfusion injury in cardiomyocytes via the activation of the ERK1/2 Mitogen-activated protein kinase (MAPK) signaling pathway and may contribute to ischemic preconditioning 68. Zhan et al 69 demonstrated that oxidative low density lipoprotein (OX-LDL) and homocysteine promoted the activation of endothelial cells, the secretion of exosomes, and the increased HSP70 content of exosomes from endothelial cells; HSP70, which further activated monocytes, lead to monocyte adhesion to endothelial cells. Exosomal metastasis-associated lung adenocarcinoma transcript 1 (MALAT1) was derived from OX-LDL-treated endothelial cells and promoted M2 macrophage polarization, which is defined as an increase in the expression of M2 macrophage markers (CD206, arginase-1, and IL-10) and decreases in the expression of an M1 macrophage marker (IL-12) 70. Hergenreider et al 71 also found that exosomes released by Krüppel-like factor (KLF) 2-transduced endothelial cells transferred to smooth muscle cells and reduced atherosclerotic lesion formation in a miRNA 143/145-dependent manner. Moreover, endothelial cell-derived miR-214-containing exosomes could inhibit senescence and promote angiogenesis in human and mouse endothelial cells 72. Njock et al 73 reported that exosomal miR-10a released from endothelial cells was taken up by monocytic cells and could inhibit inflammatory signaling via targeting the NF-κB pathway. Recently, Hou et al 74 found that laminar shear stress or exercise training directly increased miR-342-5p synthesis in endothelial cells and increased the miR-342-5p-enriched exosomes that inhibited cardiomyocyte apoptosis after myocardial ischemia/reperfusion injury by targeting Caspase 9 and JNK2; in addition, the stress improved survival signaling (p-Akt) by targeting the phosphatase gene Ppm1f.

### Smooth muscle cell-derived exosomes

Vascular smooth muscle cell-derived exosomes could mediate the transfer of KLF 5-induced miR-155 from smooth muscle cells to endothelial cells, which increases endothelial permeability and promotes atherosclerotic plaque progression 75. Ding et al 76 reported that vascular smooth muscle cells that were treated with hsa-let-7g could inhibit autophagy and apoptosis, also leading to reduced intracellular ROS generation. Moreover, exosomes have been demonstrated to play crucial roles in vascular calcification by initiating mineral deposition 77,78; exosomes also can regulate the process of vascular calcification via transporting microRNAs to recipient smooth muscle cells 78-80. An *in vivo* study showed that exosomes were found in arteries from patients with chronic kidney disease, and CD63 (a biomarker of exosomes) was observed to colocalize with calcification 81. Cytokines and growth factors were also found to promote exosome release, contributing to smooth muscle cell calcification in response to environmental calcium stress 81,82.

### Cardiomyocyte-derived exosomes

Gupta et al 33 first reported the exosomes released by cardiomyocytes, and exosomes containing HSP60 could protect cells against myocardial infarction. The elevation of HSP20 in cardiomyocytes also improved cardiac function and angiogenesis via activating exosomes biogenesis in diabetic mice 83. Moreover, cardiomyocyte-derived exosomes could regulate glycolytic flux in endothelial cells by the direct transfer of glycolytic enzymes and GLUT transporters 84. Exosomes released from hypoxic cardiomyocytes also inhibited autophagy by transferring miRNA-30a between cardiomyocytes in a paracrine manner 85. Notably, Wang et al 86 found that exosomes released by cardiomyocytes could exert an anti-angiogenic function (the inhibition of endothelial cell proliferation, migration and tube formation) in type 2 diabetic rats through the exosomal transfer of miR-320 into endothelial cells. In addition, cardiomyocyte-derived exosomes in infarcted hearts have been demonstrated to increase the injury of transplanted bone marrow mesenchymal stem cells 87. Hypoxia inducible factor-1α (HIF-1α) also initiated TNF-α expression in acute myocardial infarction, which was mediated by exosomes derived from cardiomyocytes 88.

### Dendritic cells and monocyte-derived exosomes

Gao et al 89 showed that exosomes derived from mature dendritic cells are involved in endothelial inflammation through the membrane TNF-α-mediated NF-κB pathway and that exosomes could be taken up by aortic endothelial cells and could induce inflammation and atherosclerosis. Exosomes released from primary human monocytes, dendritic cell precursors, could be taken up by endothelial cells; this contributes to endothelial dysfunction via the TLR4 and NF-κB pathways 90. The monocyte-derived exosomes mediated transfer of microRNA-150 from monocytes to endothelial cells can enhance the capillary tube formation of endothelial cells and promote angiogenesis 91. Moreover, another study 92 reported that exosomes secreted from LPS-activated macrophages affected the gene expression and differentiation of adipocyte, which might play a critical role in atherosclerosis. Chronic inflammation is a crucial factor involved in atherosclerosis, and dendritic cell and monocyte-derived exosomes promote the development and progression of coronary artery disease.

### Adipose cell-derived exosomes

Clinical study 93 showed that the imbalance of epicardial adipose tissue volume and adipocytokine was strongly related to coronary atherosclerosis. Adipose cell-derived circulating exosomes miRNAs could regulate gene expression in distant tissues 94. Adipose cell-derived exosomes also have been reported to mediate the activation of TNF-α and IL-6 in macrophages and insulin resistance via the TLR4/ Toll-interleukin-1 receptor domain-containing adaptor protein inducing interferon-β (TRIF) pathway 95. Exosomes derived from insulin resistance adipocyte in diabetic ApoE deficit mice could promote atherosclerosis and vulnerable plaque via vasa vasorum angiogenesis 96. Additionally, a recent study showed that cardiomyocytes, uptake adipose cell-derived exosomal miR-214 through clathrin-mediated endocytosis, could prevent cardiomyocyte damage after acute myocardial infarction 97. Exosomes released by adipose cell-derived MSC have been demonstrated to decrease cell adhesion molecules expression via inhibiting the MAPK and NFκB pathways and reduce atherosclerosis in LDL receptor-deficient mice 98.

### Platelet-derived exosomes

Fifteen years ago, Caby et al 99 demonstrated that blood was a physiological fluid for exosomes circulation, indicating an important role of exosomes in cell-to-cell communication through the transfer of cellular material to reach distant recipient cells. In FeCl_3_-induced murine carotid arteries, platelet-derived exosomes could suppress atherothrombotic processes by enhancing the protein ubiquitination and proteasome degradation of CD36 (a type II scavenger receptor in platelets) and by inhibiting platelet activation and thrombosis 100. Thrombin-activated platelet-derived exosomes inhibit the endothelial cell expression of intercellular adhesion molecule-1 (ICAM-1) by microRNA-223 during inflammation 101. Thrombin-stimulated platelet-derived exosomes containing miR-223, miR-339 and miR-21 inhibit platelet-derived growth factor receptor-β expression in vascular smooth muscle cells 102. Overall, platelet-derived exosomes may serve as therapeutic targets of coronary artery disease by inhibiting platelet activation, suppressing inflammation, and reducing the proliferation of smooth muscle cells.

### Exosomes from other sources

Plasma exosomes from rats and healthy volunteers exhibit cardioprotective functions against ischemia-reperfusion injury through the HSP70/TLR4 communication axis 103. Exosomes secretion after ischemic preconditioning is involved in cardioprotection by remote ischemic preconditioning, underlining the importance of exosomal transfer mechanisms in remote cardioprotection 104. Exosomal miR-29a, a key regulator of tissue fibrosis, leads to the beneficial effect of remote ischemic conditioning to improve left ventricular remodeling on chronic heart failure after acute myocardial infarction 105. Exosomal miR-144 could play a crucial role in the cardioprotection induced by remote ischemic preconditioning, and miR-144 might serve as a novel therapeutic target to decrease clinical ischemia-reperfusion injury 106. Exosomal miR-1 and miR-133a were derived from the injured myocardium 107 after acute myocardial infarction and could be transferred to adjacent myocardium to protect cardiomyocytes against hypertrophy. Moreover, Cheng et al 108 reported that urine exosomal miR-1 could be a novel urine biomarker for acute myocardial infarction. Matsumoto et al 109 demonstrated that circulating microRNAs (miR-34a, miR-192, and miR-194) were predictive indicators of ischemic heart failure via the p53 pathway in patients with acute myocardial infarction. The exosomes released from the human cardiac muscle patches (human induced-pluripotent stem cells) were shown to have cardioprotective effects that increased the survival of cardiomyocytes 110. A porcine model 111 showed that cardiosphere-derived exosomes could decrease scarring after myocardial infarction, reduce adverse remodeling and increase left ventricular ejection fraction in acute and convalescent myocardial infarctions. Exosomal miR-181b from cardiosphere-derived cells could induce macrophage polarization and reduce protein kinase C transcript levels; in addition, they exhibit a cardioprotective effect in acute myocardial infarction 112. Recently, Cheng et al 113 found that circulating exosomes carrying myocardial-miRs (miR-1, miR-208, and miR-499) were transferred selectively to peripheral organs, preferentially to the bone marrow, and that these transferred myocardial-miRs downregulated CXCR4 expression in bone marrow cells, leading to progenitor cell mobilization.

### Exosomes and potential diagnostic implication in CAD

Limited studies reported the potential diagnostic implication of exosomes in CAD 12. A previous clinical study 114 showed that serum exosomes of patients with acute coronary disease (ACS) presented with a high expression of miR-208a compared with that in healthy subjects. More importantly, patients with low miR-208a expression were associated with a significant lower mortality rate than those with high miR-208a expression (3.3% vs. 11.0%, p<0.05) during one-year follow-up. Another study 115 found that the plasma exosomes and exosomal cardiac microRNAs could be increased significantly in patients undergoing coronary artery bypass surgery for up to 48 h after surgery, meanwhile these exosomes and their microRNAs could be positively related to high sensitive cardiac troponin (cardiac biomarker).

## Conclusions and perspectives

In this review, we have discussed exosomes biogenesis, secretion, uptake, isolation and detection, and we have discussed exosomes-mediated intercellular communication in CAD. Exosomes, such as those carrying proteins, lipids, and nucleic acids, could be taken up by the recipient cells in a paracrine or endocrine manner. Exosomes released from different cell types exhibit various biological functions, either detrimental or protective, depending on the cell state and the microenvironment.

Exosomes isolation, detection and purification should be standardized and simplified to ensure that exosomes analysis and application are feasible in daily clinical practice. The underlying mechanisms of exosomes biogenesis, uptake and clearance also need to be explored deeply; then, exosomes may serve as a clinical delivery tool of novel drugs for coronary artery disease. Moreover, a detailed characterization and functional assessment of exosomes is essential to confirm the full therapeutic effect on coronary artery disease. Therefore, the clinical translation of exosomes in the diagnosis and treatment of coronary artery disease is full of hope, but it still has a long way to go.

## Figures and Tables

**Table 1:** Characterization and Classification of Extracellular Vesicles.

Characterization	Exosomes	Microvesicles	Apoptotic bodies
**Similarities**	Present in all biofluids Released from almost all types of cells Includes proteins, lipids, and nucleic acids Isolation: (ultra) centrifugation Identification: electron microscope for morphology; NTA for size and concentration; western blot for biomarker expression Intercellular communication: transferring biologic information to recipient cells		
**Size (diameter)**	~ 30-150 nm	~ 0.1-1 µm	1-5 µm
**Formation**	Fusion of MVB with cell membrane	Outward budding of the plasma membrane	Release after cell apoptosis
**Formation pathways**	ESCRT pathways; Tetraspanin or ceramide pathways	Various enzymes and mitochondrial or calcium signaling pathways (complex and unclear)	Apoptosis-related pathway
**Markers**	CD9, CD63, CD81 TSG 101, flotillin, annexin HSP70, HSP90	Integrins, selections, and other antigens from parent cells High phosphatidylserine exposure	High phosphatidylserine exposure Caspase 3, histones

NTA: nanoparticle tracking analysis; MVB: multivesicular bodies; ESCRT: endosomal sorting complex required for transport; CD: cluster differentiation; TSG: tumor susceptibility gene.

## Abbreviations

CD: cluster differentiation
CAD: coronary artery disease
CXCR4: C-X-C chemokine receptor type 4
ERK: extracellular signal-regulated kinase
ESCRT: endosomal sorting complex required for transport
HIF: hypoxia-inducible factor
HSP: heat shock protein
ICAM-1: intercellular adhesion molecule-1
ILVs: intraluminal vesicles
KLF: Krüppel-like factor
MHC: major histocompatibility complex
MSCs: mesenchymal stem cells
mTORC: mechanistic target of rapamycin complex
MAPK: mitogen-activated protein kinase
MVB: multivesicular bodies
NTA: nanoparticle tracking analysis
OX-LDL: oxidative low density lipoprotein
SMC: smooth muscle cell
SNAREs: soluble N-ethylmaleimide-sensitive factor attachment protein receptors
TEM: transmission electron microscopy
TRIF: Toll-interleukin-1 receptor domain-containing adaptor protein inducing interferon-β
TSG: tumor susceptibility gene
